# Fluorinated benzalkylsilane molecular rectifiers

**DOI:** 10.1038/srep38092

**Published:** 2016-11-29

**Authors:** Zachary A. Lamport, Angela D. Broadnax, David Harrison, Katrina J. Barth, Lee Mendenhall, Clayton T. Hamilton, Martin Guthold, Timo Thonhauser, Mark E. Welker, Oana D. Jurchescu

**Affiliations:** 1 Department of Physics, Wake Forest University, Winston Salem, NC 27109, USA; 2 Department of Chemistry, Wake Forest University, Winston Salem, NC 27109, USA; 3 Department of Chemistry, Massachusetts Institute of Technology, Cambridge, MA 02139, USA

## Abstract

We report on the synthesis and electrical properties of nine new alkylated silane self-assembled monolayers (SAMs) – (EtO)_3_Si(CH_2_)_n_N = CHPhX where n = 3 or 11 and X = 4-CF_3,_ 3,5-CF_3_, 3-F-4-CF_3_, 4-F, or 2,3,4,5,6-F, and explore their rectification behavior in relation to their molecular structure. The electrical properties of the films were examined in a metal/insulator/metal configuration, with a highly-doped silicon bottom contact and a eutectic gallium-indium liquid metal (EGaIn) top contact. The junctions exhibit high yields (>90%), a remarkable resistance to bias stress, and current rectification ratios (R) between 20 and 200 depending on the structure, degree of order, and internal dipole of each molecule. We found that the rectification ratio correlates positively with the strength of the molecular dipole moment and it is reduced with increasing molecular length.

Molecular electronics has been an area of great interest as device technology closes on the limits of the often-mentioned Moore’s law, with consumer-available technology already in the tens of nanometers range. The idea of molecular electronics was proposed as a means to surpass the challenges present in downscaling existing technologies^1^,^2^,^3^; however, commercially viable examples have yet to be introduced^4^,^5^,^6^,^7^,^8^. Since 1974, when Aviram and Ratner proposed the first rectification mechanism for a molecular structure containing a donor-acceptor pair separated by a σ-bonded tunnelling bridge, the field has witnessed spectacular growth^9^. A rectifier is a device which allows a large amount of current to pass through at one bias while restricting current flow at the opposite bias. While the above mentioned theoretical work extrapolated the inorganic solid state p-n junction miniaturized to the size of a single molecule and relied on the manipulation of the molecule’s energy levels, several different mechanisms for enhanced rectifications have subsequently been proposed^10^,^11^,^12^,^13^. Further experimental work has also demonstrated that the donor-acceptor pair may not always be necessary, and that systems with a single π-bonded component and a σ-bonded insulating chain can provide a more consistent, as well as a greater degree of, rectification^14^,^15^,^16^. Significant research effort was dedicated toward the examination of both single-molecule and molecular assemblies as rectifiers, with clear advantages exhibited by both^17^,^18^,^19^,^20^. Single molecule devices allow for a direct comparison with the theoretical calculations due to the lack of complexity in such systems. Nevertheless, Nijhuis *et al*. produced convincing arguments for the observed rectification in ferrocene-terminated alkanethiolate molecules assembled on surfaces in monolayer patterns^21^,^22^,^23^. Other self-assembled monolayers and Langmuir-Blodgett films provide ease of fabrication due to the molecule’s penchant for assembling on a well-chosen surface. This allows for the formation of many devices on a single substrate simultaneously and without the difficult alignment task present in single-molecule structures. The research to improve the rectifying behaviour of molecular diodes, however, has proceeded mostly by trial and error and a relation between the molecular structure and resulting strength of rectification has not been clearly demonstrated.

In this work, we developed nine new fluorinated benzalkylsilane molecules of varying internal molecular dipole due to the imposed length and termination, and we studied their electrical properties in relation to their chemical structure. We incorporated the self-assembled monolayers (SAMs) composed of these molecules into molecular diodes and obtained high yields and reproducible rectification ratios as high as 200, with good bias stress stability. We calculated the molecular dipole using density functional theory (DFT) and we found that the rectifying behaviour is stronger in the case of short molecules and is enhanced by the internal dipole. Our results provide evidence that the molecular structure impacts the electrical properties of molecular diodes through the internal dipole characteristic to each molecule, and may promote a rational design of compounds that can function as high-performance molecular rectifiers.

## Results

### Molecular Structures and Analysis

**Figure 1:**
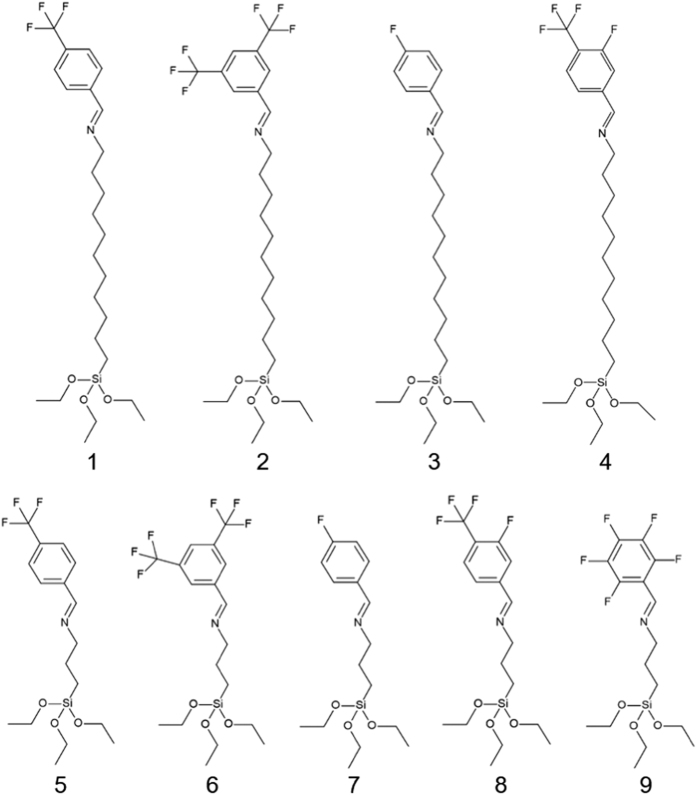
Chemical diagrams of molecules 1–9.

The examined molecules were fluorine-substituted benzaldehyde imine-terminated trialkoxysilanes, designed such that each contains a triethoxysilane group that ensures the attachment on the Si/SiO_2_ surface, an alkyl chain with either 3 or 11 CH_2_ groups, a phenyl group to facilitate the formation of an “intermolecular top-link” to enhance ordering, and a fluorine-containing head-group providing different amounts and orientations of fluorine atoms^24^. The chemical structures are included in Fig. 1, as follows: (*E*)-1-(4-(trifluoromethyl)phenyl)-*N*-(11-(triethoxysilyl)undecyl)methanimine (molecule 1), (*E*)-1-(3,5-bis(trifluoromethyl)phenyl)-*N*-(11-(triethoxysilyl)undecyl)methanimine (molecule 2), (*E*)-1-(4-fluorophenyl)-*N*-(11-(triethoxysilyl)undecyl) methanimine (molecule 3), (*E*)- 1-(3-fluoro-4-trifluoromethyl)phenyl)-*N*-(11-(triethoxysilyl)undecyl)methanimine (molecule 4), (*E*)-1-(4-(trifluoromethyl)phenyl)-*N*-(3-(triethoxysilyl)propyl)methanimine (molecule 5), (*E*)-1-(3,5-bis(trifluoromethyl)phenyl)-*N*-(3-(triethoxysilyl)propyl) methanimine (molecule 6), (*E)*-1-(4-fluorophenyl)-*N*-(3-(triethoxysilyl)propyl) methanimine (molecule 7), (*E*)- 1-(3-fluoro-4-trifluoromethyl)phenyl)-*N*-(3-(triethoxysilyl)propyl)methanimine (molecule 8) and (*E*)-1-(perfluorophenyl)-*N*-(3-(triethoxysilyl)propyl) methanimine (molecule 9). (*E*)-1-(perfluorophenyl)-*N*-(11-(triethoxysilyl)undecyl) methanimine (the long chain analogue of molecule 9) was also investigated; its properties, however, varied significantly from sample to sample, as well as for the same film, and therefore we decided not to include it in our analysis. The molecules were synthesized from amino trialkoxysilanes by using the condensation of commercially available amino alkyl triethoxysilanes with substituted benzaldehydes to prepare the precursors, following established procedures (see Fig. SI 1, Supporting Information)^25^,^26^. We will refer to molecules 1, 2, 3, and 4 as the “long molecules” (their lengths are between 2 and 2.2 nm) and molecules 5 through 9 as the “short molecules” (their lengths are between 0.9 and 1.1 nm). The length was evaluated using Spartan software, measured from the Si atom to the most distal atom of the head-group for the lowest energy conformer of each molecule.

### Device Fabrication

To examine their electrical properties, the SAMs were deposited on highly-doped, natively oxidized silicon (001) wafers using solution or vapor-based deposition, as described in the Methods section.

### Surface Analysis

In order to evaluate the quality of the SAM and the best method for assembly, water contact angle measurements were conducted using a Ramé-Hart Model 200 Contact Angle Goniometer; the results are displayed in Table 1 and in Fig. SI 2, Supporting Information. A high value of the contact angle generally coincides with a higher degree of order and/or a denser film^27^. Indeed, the contact angles measured for all the SAMs were around 70°. These values agree with the measurements performed on other fluorinated SAMs deposited on SiO_2_^28^. For reference, we have also measured the contact angle on untreated substrates, and that is displayed in the same figure. It can be observed that the contact angle for bare native SiO_2_ is <10°.

**Figure 2:**
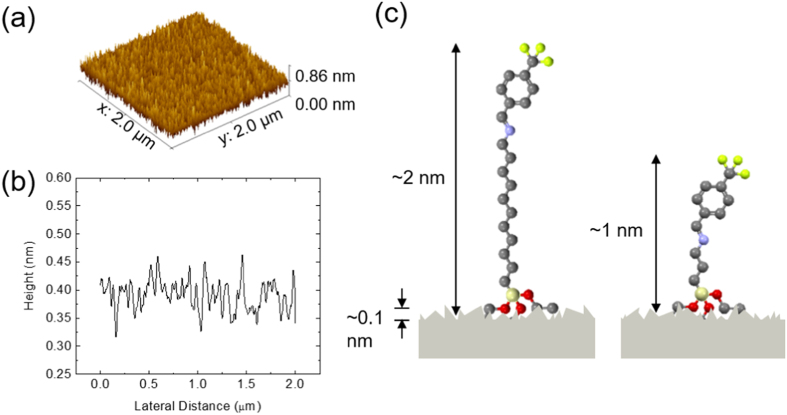
(**a**) Isometric view of a single AFM scan, (**b**) general line profile from AFM scan, and (**c**) schematic representation, to scale, of the SAM on a device substrate showing the significant difference in length for both the long (left) and short (right) molecules compared to the surface roughness.

We employed atomic force microscopy (AFM) measurements to characterize the surface roughness prior to SAM deposition. We measured a sample of 1 cm × 1 cm and imaged a surface area of approximately 2 × 2 μm in several spots using a Nanoscope IIIA AFM (Veeco Instruments), see Fig. 2. This surface is of similar quality to that obtained using template stripped or flip-chip laminated metallic electrodes^22^,^29^. The roughness value is over an order of magnitude smaller than the height of the molecules studied here, including the shortest one, and therefore it is largely inconsequential with regard to the assembly of a film, allowing for the formation of a highly ordered film when the processing parameters are carefully tuned. A graphical illustration of this can be seen in Fig. 2(c), where we schematically show the assembly of a long and a short molecule studied here and the respective difference in the length scales of the substrate and molecules.

The work function measurements on highly doped silicon were performed using a Trek model 325 electrostatic voltmeter configured for Kelvin probe measurements and calibrated using highly-ordered pyrolytic graphite (HOPG) as a standard. The calculations followed from equation 1:


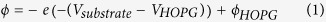


where *e* is the elementary charge, 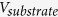 is the measured signal from the substrate (Si with a native layer of SiO_2_), 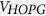 is the measured signal from HOPG, and 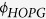 is the work function of HOPG (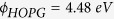)^30^. Four samples were evaluated, in at least 8 spots, and the results were consistent: we obtained a work function of 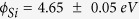 for the highly doped Si substrates with a thin layer of native oxide on their surface.

### Electrical Characterization

**Figure 3:**
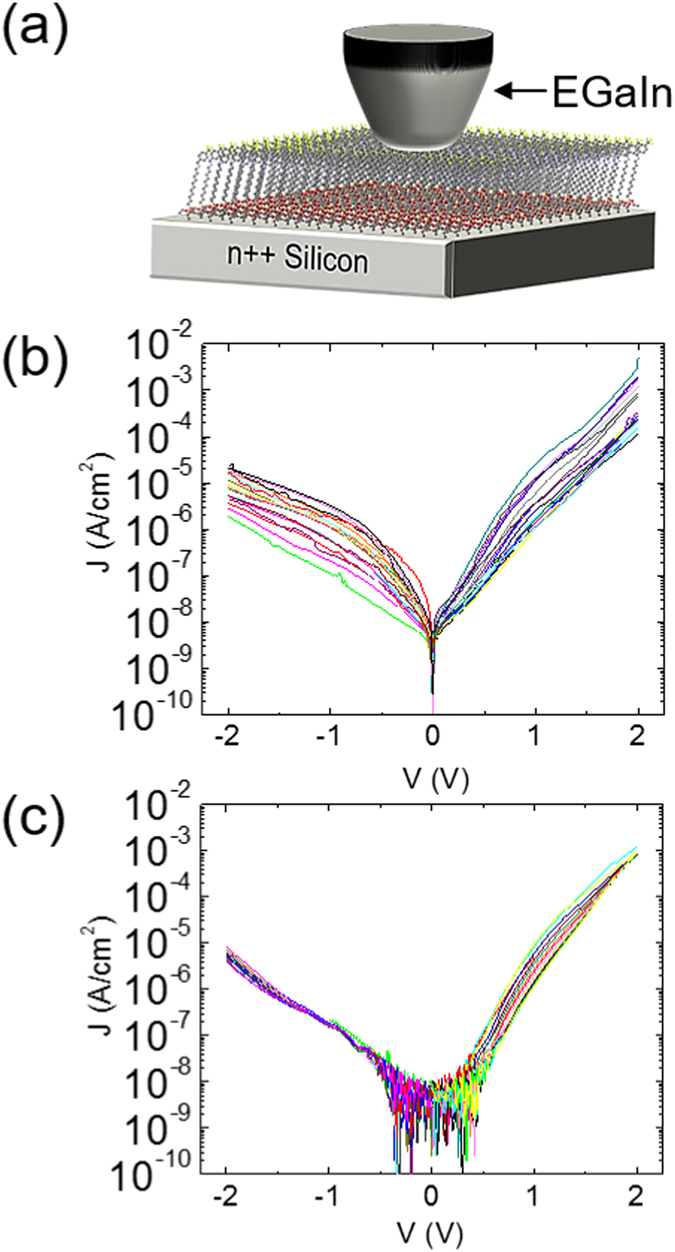
(**a**) Schematic of device structure showing a SAM sandwiched between the native SiO_2_ bottom contact and EGaIn top contact. (**b**) Current density versus the applied voltage on 25 different spots in a 1 cm × 1 cm area on a film consisting of molecule 3. (**c**) Bias stress measurements on molecule 6 over 50 consecutive measurements in a single spot.

The electrical measurements were taken on metal/SAM/metal structures using a eutectic gallium-indium (EGaIn) conical-tip top contact in which the silicon substrate was held at ground and the bias was applied to the EGaIn contact in ambient conditions. In this configuration, the forward bias condition corresponds to a positive potential applied at the fluorinated end of the molecules. The eutectic liquid metal EGaIn was utilized as a soft top-contact because its use is non-damaging, reproducible, and well-studied in this context^22^,^23^,^31^,^32^,^33^,^34^,^35^,^36^,^37^,^38^. A schematic of the device structure used in this study is included in Fig. 3(a). This figure, however, denotes an ideal structure of the device, while we are aware that our SAM layers may exhibit a lower degree of order, different orientation with respect to the substrate, and possible step-edges due to substrate imperfections, impurities, etc.

### Ab Initio Calculations

We performed *ab initio* calculations at the density functional theory level, using VASP^39^,^40^. We used the standard projector augmented wave (PAW) pseudopotentials provided by VASP along with the Perdew-Burke-Ernzerhof (PBE) exchange-correlation functional^41^,^42^. All calculations were done with a kinetic-energy cutoff of 400 eV and an energy convergence criterion of 10^−4^ eV. Calculations were performed in orthorhombic unit cells with a vacuum of at least 2 nm between periodic images. All structures were relaxed until the maximum force on any atom was less than 10 meV/angstrom. Dipole moments were calculated using VASP’s built-in functionality to calculate and correct for the dipole moment in all directions (i.e. the IDIPOL and LDIPOL tags).

### Electrical Properties of Molecular Diodes

In Fig. 3(b) we show the dependence of the current density (*J*) on the applied voltage (*V*) for one film of molecule 3, in 25 different spots. The relative standard deviation for these measurements is <1.4. Measurements on different spots on the films are consistent, suggesting that our SAMs are uniform. In addition, for a particular SAM to be considered viable as a rectifier, it must be able to withstand many repetitive measurements with minimum changes in the electrical characteristics. This could involve a variability and instability in the measured output characteristics or, in the worst-case scenario, a “breaking” of the monolayer, indicating that there is now an irreversible conductive path between the two electrodes. The good operational stability, as clearly seen in Fig. 3(c) for molecule 5, where we show the results of 50 consecutive measurements, indicates that these monolayers are robust to bias stress (the relative standard deviation is less than 0.21). The difference between Fig. 3(b) and (c) at low voltages originates from the fact that charging effects may occur when repeated measurements are taken on the same spot on the film (Fig. 3(c)), causing noise and asymmetries in the measured current. We note, however, that applying higher voltages may result in irreversible damage of the SAM film. The breakdown field in these SAMs range from approximately 20 MV/cm for molecule 9 to 50 MV/cm for molecule 5.

**Figure 4:**
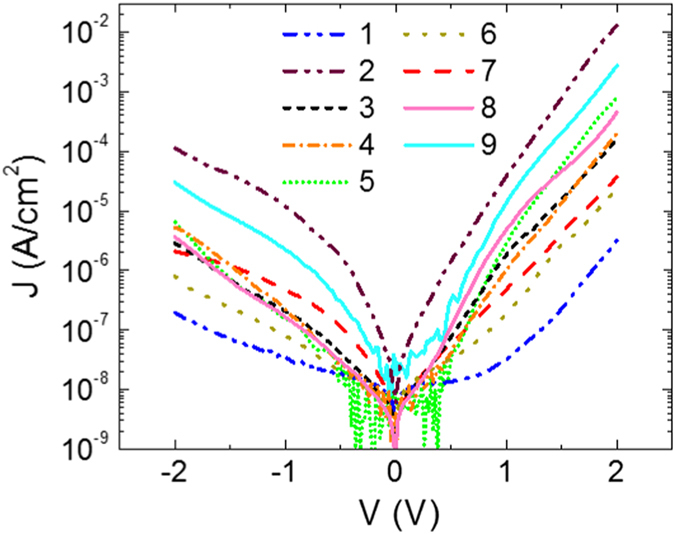
Representative current density versus applied voltage curves films of molecules 1–9.

Figure 4 shows typical current-voltage (*J-V*) characteristics for each of the 9 molecules, taken during the forward and reverse bias conditions. We measured at least 10 devices for each type of SAM, in at least 25 spots each, and obtained similar results. As expected, the magnitude of the measured current depends on the molecular structure, with the long molecules generally exhibiting lower conductivities and less pronounced asymetries between the forward and reverse bias conditions.

## Discussion

We define the rectification ratio, R, as the ratio between the *J* value during the forward bias measurement, (V = +2 V) and the *J* value during the reverse bias measurement, (V = −2 V):


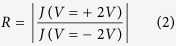


**Figure 5:**
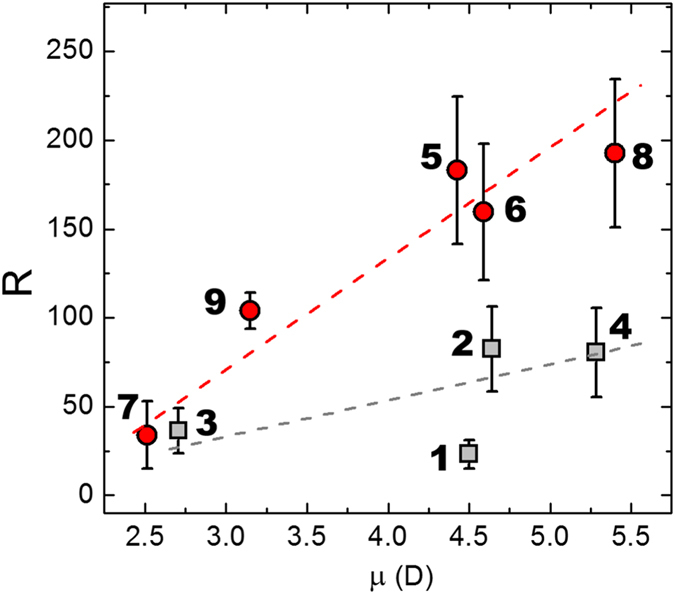
Rectification ratio vs. the dipole moment of each molecule. In red we show the results corresponding to the short molecules and in grey for the long molecules.

In Table 1 we list the R values obtained for the nine molecules. In order to relate the electrical properties with the molecular structure, we determined the electrical dipole of each molecule using DFT calculations; results are listed in Table 1. In Fig. 5 we plot the dependence of R on the molecular dipole for all the molecules investigated in this study. Results obtained for short molecules are included in red, while those for long ones are shown in grey. First, note the high value of R obtained for molecule 5 and 8, R_5_ = 183 ± 41 and R_8_ = 192 ± 42. These values are on par with some of the highest reported in the literature, with a thiophene-1,1-dioxide oligomer exhibiting an R of ~200, a ferrocene-alkanethiolate with an R = 150, and ferrocene placed on a monolayer of β-cyclodextrin demonstrating an R of 170^15^,^33^,^43^. We note, however, that larger values can also be obtained^44^,^45^. Nevertheless, our molecules have the additional advantage of being very low-cost since the precursors are common organic molecules, being easy to synthesize, and requiring minimal purification steps. This figure also denotes that a stronger rectification is achieved in the case of short molecules, in agreement with previous reports^46^,^47^. Another key point that can be observed from Fig. 5 is that for molecules of similar length, the rectification ratio correlates positively with the internal dipole moment of each molecule. This trend can originate from the fact that the molecular dipole creates a local electric field proportional to the magnitude of the dipole moment. This field contributes to the total net field experienced by the molecule under external bias, modifying the shape of the tunnelling barrier, and, consequently, of the current magnitude. Although a detailed interpretation of this dependence is beyond the scope of this manuscript, the results suggest that for molecules of similar length, the rectification increases with the molecular dipole, regardless of structural details of each compound. The trend is monotonic for the case of short molecules, and we note that there is a spread in our data for the long molecules. On one hand, this may originate from variations in molecular orientation and the degree of order of the SAMs on the surfaces, which results from the competition between intermolecular interactions of the SAM molecules and molecule-substrate interactions. We attempted to characterize the ordering of the SAMs through polarized modulated-infrared reflection absorption spectroscopy (PM-IRRAS) and X-ray reflectivity measurements, both of which were unsuccessful due to the small size of the molecular layers. On the other hand, local molecular torsions and changes in the conformation were shown to significantly impact the value of the rectification ratio^48^,^49^. Several other factors may contribute in the observed asymmetries. First, the two contacts are not perfectly symmetric: a strong, Si-O covalent bond is established between the SAM and the bottom contact, while the top contact interacts with the SAM via a weak, van der Waals bond established between the end-group of the SAM and the Ga_2_O_3_ layer formed at the surface of the EGaIn electrode^22^. The strength of this bond, and therefore the rate of charge tunnelling, may vary with changing the chemistry of the SAM fluorinated functional group. In addition, these contacts have slightly different work-functions (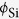
= 4.65 ± 0.05 eV, as determined from Kelvin probe measurements described above, while 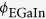 = 4.1–4.2 eV^50^), which impact the injection of charge carriers. Second, additional dipole moments may be formed through band-bending when physical contact is established between the SAM and electrodes. Nevertheless, the dependence is clearly distinguished and this suggests that tailoring the internal molecular dipole via molecular design may be a powerful tool in controlling the rectification ratios in molecular diodes. This result may seem to contradict that of Yoon and collaborators, who showed that the rectification behaviour of molecular diodes is independent of their molecular dipole^12^. In that study, however, the molecules had various lengths, and therefore the dependence may have been masked. Further studies, focusing on the examination of properties of different classes of compounds—including compounds with dipole moments in opposite directions—as well as studies on SAMs of different anchoring and terminal groups would clarify the generality and limitations of this method.

## Conclusions

In conclusion, we have designed, synthesized, and measured nine new rectifying self-assembled monolayers of different lengths and polar terminations. We found that these fluorinated benzalkylsilane molecules exhibit high yield when incorporated in molecular diodes, coupled with robust rectification ratios ranging from 20–200, depending on their structure. In addition, they show good uniformity over large surface areas and excellent bias stress stability. We found an increase in the rectification strength with enhancing the internal molecular dipole of each molecule. Our results suggest that the electrical properties of molecular diodes can be controlled by tailoring the molecular structures of the constituent molecules. This is a significant step toward controlling the performance of molecular rectifiers through manipulating specific structure-property relationships for use in macroscopic electronics as diodes, half-wave rectifiers, AC/DC converters, or other charge-restricting elements.

## Methods

The molecules were synthesized from amino trialkoxysilanes by using standard procedures (see Fig. SI 1). With the exception of one imine, see Fig. SI 1, the yields for all of these reactions were in the 68–87% range and the only purification of the products required after the reaction was filtration and removal of residual solvent under high vacuum. The new compounds were characterized by ^1^H and ^13^C nuclear magnetic resonance (NMR) and elemental analysis or high-resolution mass spectrometry (see Supporting Information for synthesis and characterization details).

The SAMs were deposited on highly-doped silicon wafers with native SiO_2_ formed at their surface by either solution or vapor-based techniques. The substrates were cleaned in hot acetone, then hot isopropanol (IPA), followed by a 10 minute exposure to UV-ozone and subsequent rinse in DI water before being dried in a stream of nitrogen. The UV-ozone step serves to both remove organic contaminants and to increase the density of hydroxyl groups on the surface. The monolayers were formed in a nitrogen (<0.1 ppm H_2_O, <0.1 ppm O_2_) glovebox. We found that for the long SAMs (molecules 1, 2, 3, and 4) self-assembly from a 4 mMol solution in chloroform at 30 °C provided the films of highest quality. The short SAMs (molecules 5 through 9) were amenable to deposition by vapor treatment, where the silicon wafer was placed into a sealed jar along with 11 μL of the pure SAM compound at 30 °C. Both methods typically took 18 to 24 hours. To remove the excess adsorbed molecules on the monolayer surface, the samples were thoroughly rinsed in chloroform followed by IPA and then immediately dried in a stream of nitrogen.

The conical-tip EGaIn contact, which was used as a top soft contact for our molecular rectifiers, was created using a Micromanipulator probe holder, and a 0.5 mm diameter Micromanipulator probe tip. EGaIn was placed onto a sacrifical copper strip into which the probe tip was slowly lowered and then raised with the aid of the contact angle goniometer.

## Additional Information

**How to cite this article**: Lamport, Z. A. *et al*. Fluorinated benzalkylsilane molecular rectifiers. *Sci. Rep.* **6**, 38092; doi: 10.1038/srep38092 (2016).

**Publisher's note:** Springer Nature remains neutral with regard to jurisdictional claims in published maps and institutional affiliations.

## Supplementary Material

Supplementary Information

## Figures and Tables

**Table 1:** Contact angles, average rectification ratios, and dipole moments of molecules 1–9.

Molecule	Contact Angle (°)	R	μ (D)
1	74	23 ± 8	4.50
2	72	82 ± 24	4.64
3	70	36 ± 13	2.71
4	79	80 ± 25	5.29
5	66	183 ± 41	4.42
6	74	159 ± 38	4.59
7	77	34 ± 19	2.48
8	78	192 ± 42	5.40
9	70	104 ± 10	3.21

(R values included with standard deviation).
