# Supplementary material for: Fluorinated benzalkylsilane molecular rectifiers
Source: Sci Rep. 2016 Nov 29;6:38092. doi: 10.1038/srep38092 (PMC5126687; doi:10.1038/srep38092)
Supplement: Supplementary Information [file srep38092-s1.pdf]

Fluorinated benzalkylsilane molecular rectifiers

Supplementary Information

Zachary A. Lamport[a], Angela D. Broadnax[b], David Harrison[a], Katrina J. Barth[a], Lee Mendenhall[b], Clayton T. Hamilton[b], Martin Guthold[a], Timo Thonhauser[a,c], Mark E. Welker[b], and Oana D. Jurchescu[a]

[a]Department of Physics, Wake Forest University, Winston-Salem, NC 27109

[b]Department of Chemistry, Wake Forest University, Winston-Salem, NC 27109

[c]Department of Chemistry, Massachusetts Institute of Technology, Cambridge, MA 02139, USA

Chemical Synthesis

The proton nuclear magnetic resonance (^{1}H NMR) spectra were obtained using a Bruker Avance 300 MHz spectrometer operating at 300.1 MHz or a Bruker Avance 500 MHz spectrometer operating at 500.1 MHz. ^{13}C NMR spectra were obtained using a Bruker Avance 300 MHz spectrometer operating at 75.5 MHz. ^{1}H and ^{13}C NMR spectra were referenced to the residual proton or carbon signals of the respective deuterated solvents. All elemental analyses were performed by Atlantic Microlabs Inc., Norcross, GA. High-resolution mass spectrometry was performed at the Mass Spectrometry Facility at Northwestern University, Evanston, IL.

All reactions were carried out under an atmosphere of nitrogen. Aminoundecyltriethoxysilane was purchased from Gelest and used as received.

Deuterated solvents were purchased from Cambridge Isotope Laboratories and dried over molecular sieves. Aminopropyltriethoxysilane and the benzaldehydes were purchased from Aldrich Chemical Company and used as received. Anhydrous sodium sulfate and HPLC grd hexane were purchased from Fisher/Acros and used as received.

(*E*)-1-(4-trifluoromethyl)phenyl)-*N*-(11-(triethoxysilyl)undecyl)methanimine (**1**). Anhydrous Na_2SO_4 (4.0 g) was added to a solution of 4-trifluoromethylbenzaldehyde (0.350 g, 2.01 mmol) in HPLC grade hexane (7 mL). A solution of aminoundecyltriethoxysilane (AUDTES) (0.738 g, 2.211 mmol) in HPLC grade hexane (7 mL) was added dropwise with stirring to the resulting mixture over 12 min. After 3 h, some additional precipitate was noted, the solution was decanted, the residue was rinsed with additional hexane (5 mL) and hexane was removed in vacuo. Compound **1** was isolated as a clear liquid (0.719 g, 1.47 mmol, 73%): ^{1}H NMR (300 MHz, $CDCl_3$) δ 8.31 (s, 1H), 7.83 (d, J = 6 Hz, 2H), 7.66 (d, J = 6 Hz, 2H), 3.81 (q, J = 6 Hz, 6H), 3.63 (t, J = 6 Hz, 2H), 1.70 (m, 2H), 1.61-1.26 (m, 18H) 1.22 (t, J = 6 Hz, 9H), 0.62 (t, J = 9 Hz, 2H); ^{13}C NMR (75.47 MHz, $CDCl_3$) δ 159.21, 139.46, 132.01 (q, J = 32.3 Hz), 128.20, 125.53 (q, J = 3.8 Hz), 123.96 (q, J = 270 Hz), 61.90, 58.29, 33.21, 30.79, 29.63, 29.58, 29.54, 29.44, 29.26, 27.36, 22.76, 18.31, 10.39. Elem. Anal. Calcd. for $C_{25}H_{42}F_3NO_3Si$: C, 61.32; H, 8.65; Found: C, 61.56; H, 8.84.

(*E*)- 1-(3,5-bistrifluoromethyl)phenyl)-*N*-(11-(triethoxysilyl)undecyl)methanimine (**2**). Anhydrous Na_2SO_4 (4.0 g) was added to a solution of 3,5-bis(trifluoromethyl)benzaldehyde (0.121 g, 0.500 mmol) in HPLC grade hexane (7 mL).

A solution of AUDTES (0.172 g, 0.515 mmol) in HPLC grade hexane (7 mL) was added dropwise with stirring to the resulting mixture over 12 min. After 3 h, some additional precipitate was noted and the solution was decanted, the residue was rinsed with additional hexane (5 mL) and hexane was removed in vacuo. Compound **2** was isolated as a light yellow liquid (0.208 g. 0.373 mmol, 75%). ^{1}H NMR (300 MHz, $CDCl_3$) δ 8.35 (s, 1H), 8.18 (s, 2H), 7.90 (s, 1H), 3.81 (q, *J* = 7.0 Hz, 6H), 3.66 (t, *J* = 6.9 Hz, 2H), 1.70 (q, *J* = 6.9 Hz, 2H), 1.47 – 1.11 (m, 25H), 0.72 – 0.51 (m, 2H). ^{13}C NMR (75 MHz, $CDCl_3$) δ 155.29, 136.16, 130.61, 130.16, 129.94 (q, *J* = 33.6 Hz), 125.71, 122.85, 121.56, 121.04 (q, *J* = 272.7 Hz), 59.66, 56.16, 31.07, 28.55, 27.51, 27.43, 27.40, 27.29, 27.12, 25.23, 20.63, 16.17, 8.28. Elem. Anal. Calcd. for $C_{26}H_{41}NO_3F_6Si$: C, 56.00; H, 7.41; Found: C, 55.95; H, 7.35.

(*E*)- 1-(4-fluoro)phenyl)-*N*-(11-(triethoxysilyl)undecyl)methanimine (**3**).

Anhydrous Na_2SO_4 (1.33 g) was added to a solution of 4-fluorobenzaldehyde (0.083 g, 0.670 mmol) in hexane (2.3 mL). A solution of AUDTES (0.245 g, 0.737 mmol) in hexane (2.3 mL) was added dropwise with stirring to the resulting mixture over 5 min. After 3 h, some additional precipitate was noted, the solution was decanted, the residue was rinsed with additional hexane (5 mL) and hexane was removed in vacuo. Compound **3** was isolated as a light yellow liquid (0.136 g, 0.310 mmol, 46%). ^{1}H NMR (500 MHz, $CDCl_3$) δ 8.23 (s, 1H), 7.71 (dd, *J* = 8.4, 5.8 Hz, 2H), 7.08 (t, *J* = 8.6 Hz, 2H), 3.81 (q, *J* = 7.0 Hz, 6H), 3.58 (t, *J* = 7.0 Hz, 2H), 1.77 – 1.58 (m, 2H), 1.48 – 1.14 (m, 25H), 0.69 – 0.55 (m, 2H). ^{13}C NMR (126 MHz, $CDCl_3$) δ 164.14 (d, *J* = 250.3 Hz), 132.68 (d, *J* = 3.0 Hz), 129.83 (d, *J* = 8.6 Hz), 115.61 (d, *J* = 21.8 Hz), 77.27, 77.01, 76.76, 61.71, 58.29,

33.20, 30.92, 29.63, 29.59, 29.54, 29.45, 29.26, 27.36, 22.76, 18.31, 10.40. Elem. Anal. Calcd for $C_{24}H_{42}FNO_3Si$: C, 65.56; H, 9.63. Found: C, 65.84; H, 9.60.

(*E*)- 1-(3-fluoro-4-trifluoromethyl)phenyl)-*N*-(11-(triethoxysilyl)undecyl)methanimine (**4**). Anhydrous Na_2SO_4 (4.0 g) was added to a solution of 3-fluoro-4-trifluoromethyl)benzaldehyde (0.096 g, 0.500 mmol) in HPLC grade hexane (7 mL). A solution of AUDTES (0.172 g, 0.515 mmol) in HPLC grade hexane (7 mL) was added dropwise with stirring to the resulting mixture over 12 min. After 3 h, some additional precipitate was noted and the solution was decanted, the residue was rinsed with additional hexane (5 mL) and hexane was removed in vacuo. Compound **4** was isolated as a clear liquid (0.237 g. 0.467 mmol, 93%). ^{1}H NMR (300 MHz, $CDCl_3$) δ 8.26 (s, 1H), 7.62 (m, 2H), 3.81 (q, J = 7.0 Hz, 6H), 3.64 (t, J = 6.9 Hz, 2H), 1.68 (q, J = 6.9 Hz, 2H), 1.47 – 1.11 (m, 25H), 0.62 (m, 2H). ^{13}C NMR (75 MHz, $CDCl_3$) δ 158.0 (dq, J = 256, 2.1 Hz), 157.9 (d, J = 2.0 Hz), 142.2 (d, J = 7.6 Hz), 127.4 (qd, J = 4.6, 1.4 Hz), 123.6 (d, J = 3.6 Hz), 122.4 (qd, J = 273, 1.2 Hz), 119.7 (dq J = 32.9, 12.6 Hz) 115.5 (d, J = 21.5 Hz), 61.74, 58.27, 33.18, 30.68, 29.61, 29.55, 29.51, 29.40, 29.24, 27.33, 22.75, 18.28, 10.39. Elem. Anal. Calcd. for $C_{25}H_{41}NO_3F_4Si$: C, 59.15; H, 8.14; Found: C, 59.15; H, 8.08.

(E)- 1-(4-trifluoromethylphenyl)-N-(3-(triethoxysilyl)propyl)methanimine (**5**). Anhydrous Na_2SO_4 (3.0 g) was added to a solution of 4-trifluoromethylbenzaldehyde (0.235 g, 1.348 mmol) in HPLC grade hexane (5 mL). A solution of aminopropyltriethoxysilane (APTES) (0.328 g, 1.483 mmol) in HPLC grade hexane (5 mL) was added dropwise with

stirring to the resulting mixture over 10 min. After 1.5 h, some additional precipitate was noted, the solution was decanted, the residue was rinsed with additional hexane (5 mL) and hexane was removed in vacuo. Compound **5** was isolated as a clear liquid (0.422 g, 1.12 mmol, 83%): ^{1}H NMR (300 MHz, $CDCl_3$) δ 8.30 (s, 1H), 7.82 (d, J = 6 Hz, 2H), 7.64 (d, J = 6 Hz, 2H), 3.83 (q, J = 6 Hz, 6H), 3.64 (t, J = 6 Hz, 2H), 1.85 (m, 2H), 1.24 (t, J = 6 Hz, 9H), 0.68 (t, J = 9 Hz, 2H); ^{13}C NMR (75.47 MHz, $CDCl_3$) δ 159.37, 139.48, 131.97 (q, J = 19.5 Hz), 128.17, 125.45, 123.94 (q, J = 162 Hz), 64.22, 58.31, 24.18, 18.21, 8.03. HRMS (APCI-ion trap) m/z: $[M + H]^+$ Calcd for $C_{17}H_{27}O_3NF_3SiH$ 378.1707; Found 378.1704.

(E)- 1-(3,5-bistrifluoromethyl)phenyl)-*N*-(3-(triethoxysilyl)propyl)methanimine (**6**). Anhydrous Na_2SO_4 (3.0 g) was added to a solution of 3,5-bistrifluoromethylbenzaldehyde (0.242 g, 1.00 mmol) in hexane (5 mL). A solution of APTES (0.244 g, 1.10 mmol) in hexane (5 mL) was added dropwise with stirring to the resulting mixture for 5 min. After 1.5 h, with presence of hydrolyzed product precipitated, the solution was decanted, and hexane was removed in vacuo. Compound **6** was isolated as a clear liquid (0.376 g, 0.845 mmol, 85%). ^{1}H NMR (300 MHz, Chloroform-*d*) δ 8.35 (s, 1H), 8.18 (s, 2H), 7.91 (s, 1H), 3.84 (q, *J* = 7.0 Hz, 6H), 3.68 (td, *J* = 6.9, 1.1 Hz, 2H), 1.93 – 1.78 (m, 2H), 1.23 (t, *J* = 7.0 Hz, 9H), 0.73 – 0.65 (m, 2H). ^{13}C NMR (75 MHz, $CDCl_3$) δ 157.71, 138.26, 132.07 (q, *J* = 33.6 Hz), 123.18 (q, *J* = 272.6 Hz), 127.88, 123.72 (hept, *J* = 3.8 Hz), 64.17, 58.44, 24.12, 18.31, 8.12. HRMS (APCI-ion trap) m/z: $[M + H]^+$ Calc for $C_{18}H_{25}O_3NF_6SiH$ 446.1586; Found 446.1583

(E)- 1-(4-fluorophenyl)-N-(3-(triethoxysilyl)propyl)methanimine (7). Anhydrous Na_2SO_4 (3.0 g) was added to a solution of 4-monofluorobenzaldehyde (0.150 g, 1.209 mmol) in HPLC grade hexane (5 mL). A solution of APTES (0.294 g, 1.330 mmol) in HPLC grade hexane (5 mL) was added dropwise with stirring to the resulting mixture over 10 min. After 1.5 h, some additional precipitate noted, the solution was decanted, the residue was rinsed with additional hexane (5 mL) and hexane was removed in vacuo. Compound **7** was isolated as a light yellow liquid (0.345 g, 1.05mmol, 87%): ^{1}H NMR (300 MHz, $CDCl_3$) δ 8.23 (s, 1H), 7.70 (t, J = 6 Hz, 2H), 7.08 (t, J = 6 Hz, 2H), 3.82 (q, J = 6 Hz, 6H), 3.59 (t, J = 6 Hz, 2H), 1.82 (m, 2H), 1.22 (t, J = 6 Hz, 9H), 0.67 (t, J = 9 Hz, 2H); ^{13}C NMR (75.47 MHz, $CDCl_3$) δ 164.02 (d, J = 248.3 Hz), 159.49, 132.73, 129.93, 115.62 (d, J = 21.8 Hz), 64.18, 58.39, 24.29, 18.32, 8.10. HRMS (APCI-ion trap) m/z: $[M + H]^+$ Calc for $C_{16}H_{27}FNO_3Si$ (M +) calcd: 328.1744; obs: 328.1748.

(E)- 1-(3-fluoro-4-trifluoromethyl)phenyl)-N-(3-(triethoxysilyl)propyl)methanimine (**8**). Anhydrous Na_2SO_4 (3.0 g) was added to a solution of 3-fluoro-4-trifluoromethyl)benzaldehyde (0.384 g, 1.00 mmol) in HPLC grade hexane (5 mL). A solution of APTES (0.244 g, 1.10 mmol) in HPLC grade hexane (5 mL) was added dropwise with stirring to the resulting mixture over 10 minutes. After 1.5 h, the solution was decanted and hexane was removed in vacuo. Compound (**8**) was isolated as a clear liquid (0.342 g. 0.865 mmol, 87%). ^{1}H NMR (300 MHz, Chloroform-*d*) δ 8.27 (s, 1H), 7.67 – 7.53 (m, 3H), 3.83 (q, *J* = 7.0 Hz, 6H), 3.65 (t, *J* = 6.5 Hz, 2H), 1.89 – 1.79 (m, 2H), 1.23 (t, *J* = 7.0 Hz, 9H), 0.70 – 0.64 (m, 2H). ^{13}C NMR (126 MHz, $CDCl_3$) δ 159.9 (dq, *J* = 256, 2.1 Hz), 158.2 (d, *J* = 2.0 Hz), 142.2 (d, *J* = 7.7 Hz), 127.3 (qd, *J* = 4.6, 1.4

Hz), 123.7 (d, J = 3.6 Hz), 122.5 (qd, J = 270, 1.2 Hz), 119.7 (dq J = 32.9, 12.6 Hz) 115.4 (d, J = 21.5 Hz), 64.09, 58.37, 24.09, 18.25, 8.05. HRMS (APCI-ion trap) m/z: $[M + H]^+$ Calc for $C_{17}H_{25}O_3NF_4SiH$ 396.1618; Found 396.1612

(E) -1-(pentafluorophenyl)-N-(3-(triethoxysilyl)propyl)methanimine (**9**). Anhydrous Na_2SO_4 (3.0 g) was added to a solution of pentafluorobenzaldehyde (0.133 g, 0.678 mmol) in hexane (8 mL). A solution of APTES (0.150 g, 0.678 mmol) in hexane (8 mL) was added dropwise with stirring to the resulting mixture over 10 min. After 1 h, additional precipitate was noted and the solution was decanted, the residue was rinsed with additional hexane (5 mL) and hexane was removed in vacuo. Compound **9** was isolated as a light yellow liquid (0.183 g, 0.458 mmol, 68%). ^{1}H NMR (500 MHz, $CDCl_3$) δ 8.38 (s, 1H), 3.83 (q, J = 7.0 Hz, 6H), 3.70 (t, J = 6.7 Hz, 2H), 1.93 – 1.75 (m, 2H), 1.23 (t, J = 7.0 Hz, 9H), 0.71 – 0.59 (m, 2H). ^{13}C NMR (126 MHz, $CDCl_3$) δ 149.16 (p, J = 2.5 Hz), 145.70 (ddtd, J = 256.0, 11.5, 7.6, 3.9 Hz), 141.85 (dtt, J = 256.9, 13.7, 5.1 Hz), 137.6 (dtdd, J = 253.1, 14.1, 6.0, 2.0 Hz), 111.30 (td, J = 12.0, 4.2 Hz), 65.77, 58.39, 24.03, 18.27, 7.90. HRMS (APCI-ion trap) m/z: $[M + H]^+$ Calc for $C_{16}H_{22}O_3NF_5SiH$ 400.1362; Found 400.1353.

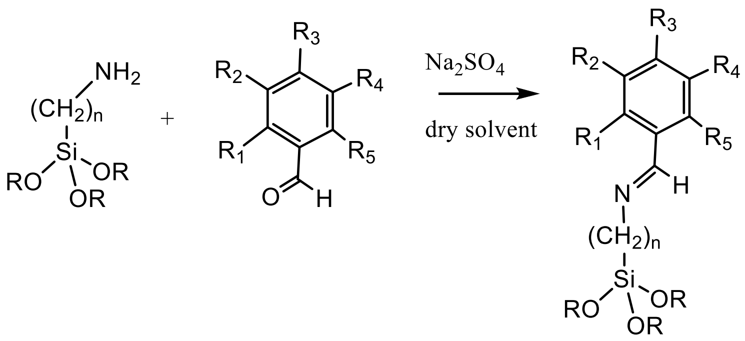

1 R = Et, n = 11, $R_3 = CF_3$, $R_{1, 2, 4, 5}$ = H, 73%

2 R = Et, n = 11, $R_2 = R_4 = CF_3$, $R_{1, 3, 5}$ = H, 75%

3 R = Et, n = 11, R_3 = F, $R_{1, 2, 4, 5}$ = H, 46%

4 R = Et, n = 11, $R_3 = CF_3$, R_2 = F, $R_{1, 4, 5}$ = H, 93%

5 R = Et, n = 3, $R_3 = CF_3$, $R_{1,2,4,5}$ = H, 82%

6 R = Et, n = 3, $R_2 = R_4 = CF_3$, $R_{1,3,5}$ = H, 85%

7 R = Et, n = 3, R_3 = F, $R_{1,2,4,5}$ = H, 87%

8 R = Et, n = 3, $R_3 = CF_3$, R_2 = F, $R_{1, 4, 5}$ = H, 87%

9 R = Et, n = 3, R_1-R_5 = F, 68%

Figure SI 1. Synthesis of molecules 1-9.

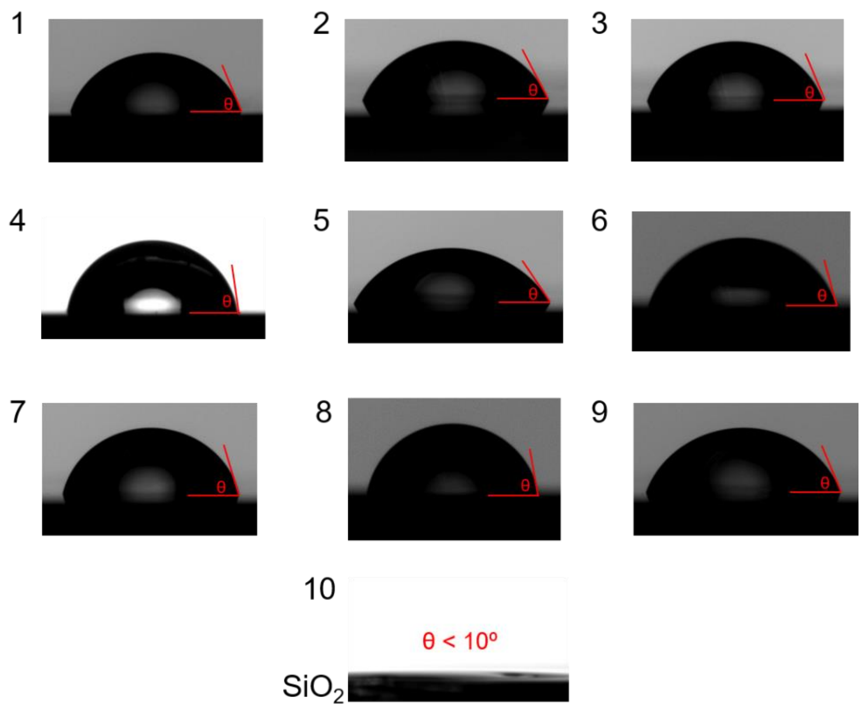

Figure SI 2. Contact angle measurements conducted on monolayers of molecules 1-9 assembled on native SiO_2. The contact angle of clean native SiO_2 is shown for reference in panel 10.
